# Maximising mosquito collections from barrier screens: the impacts of physical design and operation parameters

**DOI:** 10.1186/s13071-019-3291-4

**Published:** 2019-01-14

**Authors:** Edgar J. M. Pollard, Tanya L. Russell, Thomas R. Burkot

**Affiliations:** Australian Institute of Tropical Health and Medicine, James Cook University, QLD, Cairns, 4870 Australia

**Keywords:** Outdoor mosquito collections, *Anopheles farauti*, Barrier screen, Mosquito movement, Passive tool

## Abstract

**Background:**

Traditional methods for collecting outdoor resting mosquitoes are generally inefficient with relatively low numbers caught per unit effort. The barrier screen, designed to intercept mosquitoes as they fly between areas where blood meals are obtained and oviposition sites where eggs are laid, was developed in 2013 as a novel method of sampling outdoor mosquito populations. Barrier screens do not use an odorant lure and are thus a non-mechanical, simple, low maintenance and passive sampling method for use, even in isolated locations.

**Methods:**

To maximise mosquito collections from barrier screens, multiple Latin square 3 × 3 experiments were conducted in Smithfield, Queensland, Australia. Parameters of barrier screens were varied including the effects of construction materials (net weight and colour), screen design and frequency of inspections.

**Results:**

Significantly more mosquitoes were collected on simple dark coloured screens of 50% or 70% shading weight with collections every 30 min. Sixty percent of mosquitoes were found on barrier screens within 60 cm of the ground.

**Conclusions:**

The barrier screen is a relatively new adaptable tool that can answer a number of behavioural, ecological and epidemiological questions relevant for the surveillance and basic understanding of the movement and resting habits of mosquitoes by sex or physiological status. This method has demonstrated robustness in collecting a wide range of mosquito species as well as flexibility in where barrier screens can be deployed to explore mosquito movements within rural and peri-domestic environments.

**Electronic supplementary material:**

The online version of this article (10.1186/s13071-019-3291-4) contains supplementary material, which is available to authorized users.

## Background

Mosquito sampling using long-range odorant lures (including human landing catches) give useful insights into mosquito densities attracted to fixed locations [1] but little work has been done to understand the movement of mosquitoes between locations. Observing insects in their natural flight patterns without influencing their behaviour requires using capture methods with minimal long-distance attractants [2]. Almost all sampling techniques are prone to biases and different techniques will be better suited to collecting different subsets of insect populations [3]. Sampling techniques without odorant attractants (hereafter referred to as passive tools) are useful for representative sampling of entire populations, e.g. males and females of all physiological states (unfed, sugar-fed, blood-seeking females, recently engorged females as well as gravid females seeking oviposition sites). Often the utility of traps is constrained by requirements for attractants or trap designs (size) or power requirements for light or fans. Passive tools without moving parts or power requirements are simpler, cheaper, more robust and easier to deploy in isolated areas.

There are two functional types of passive tools; tools that provide estimates of resting mosquito numbers and tools that infer mosquito movement. Passive tools that target resting mosquitoes include pit shelters, resting pots and boxes such as the sticky resting box [4]. Common passive methods for collecting mosquitoes and inferring movement patterns include malaise traps, ramp traps, stationary nets and sticky traps [3]. The malaise trap was one of the first passive tools for insects [5] and is advantageous because it can be used in remote locations, in almost any weather condition (with the exception of high winds), where mosquito densities are high, and a human collector is not required. While the malaise trap can capture insects approaching from all directions, it generally does not allow the direction of flight to be determined. Ramp traps were invented by Gillies [6] in 1969 to study directional mosquito movement. The ramp trap guides mosquitoes into a collection chamber by a ramp with the direction of mosquito flight inferred by the orientation of the trap as insects can only enter the trap from a single direction. However, some mosquito species respond visually, either positively or negatively, to the ramp trap, thus potentially biasing the estimates of species densities [1]. Stationary nets are similar to the ramp trap but are made of nylon netting in a pyramid shape which allows flying mosquitoes to enter a large opening into a collecting sleeve [7]. The stationary net is usually oriented to capture mosquitoes flying in a single direction. Sticky traps use a sticky surface to immobilize mosquitoes [1] with movement inferred from the direction the trap is facing.

Collection of outdoor blood-fed resting mosquitoes can be extremely difficult and time-consuming [3]. The barrier screen is the newest passive attempt at collecting outdoor resting mosquitoes [8]. The barrier screen was developed to determine the frequency of mosquito blood-feeding on different host species by capturing an unbiased sample of blood-fed mosquitoes outdoors. The barrier screen is a passive tool that intercepts mosquitoes as they fly while tracking odorant cues of blood meal sources, resting, oviposition sites, swarming sites or sugar sources and allows information on the flight direction of the mosquitoes collected to be inferred from the side of the screen on which the mosquito was collected in relation to the proximity of houses, larval habitats, sugar sources and likely resting sites [8]. Barrier screens have been constructed from a variety of durable materials, such as shade-cloth, made from materials including polyvinylchloride-coated polyester, polyethylene and cotton [8]. Flying mosquitoes will encounter the screen and temporarily rest. When resting on the barrier screen, mosquitoes are easily visible and can be collected using aspirators. The impact on numbers of mosquitoes collected on barrier screens as a function of the barrier screen materials and design has not been evaluated.

To maximise mosquito numbers collected using barrier screens, different attributes of barrier screen construction (e.g. cloth weight, cloth colour, barrier design and inspection frequency) were systematically evaluated by comparing the numbers of mosquitoes captured when each individual attribute was employed. Optimising barrier screens will facilitate this tool to be utilised to better understand the natural distribution and movements of mosquitoes in time and space and thus to better inform how to monitor and control mosquitoes.

## Methods

### Study site

**Fig. 1:**
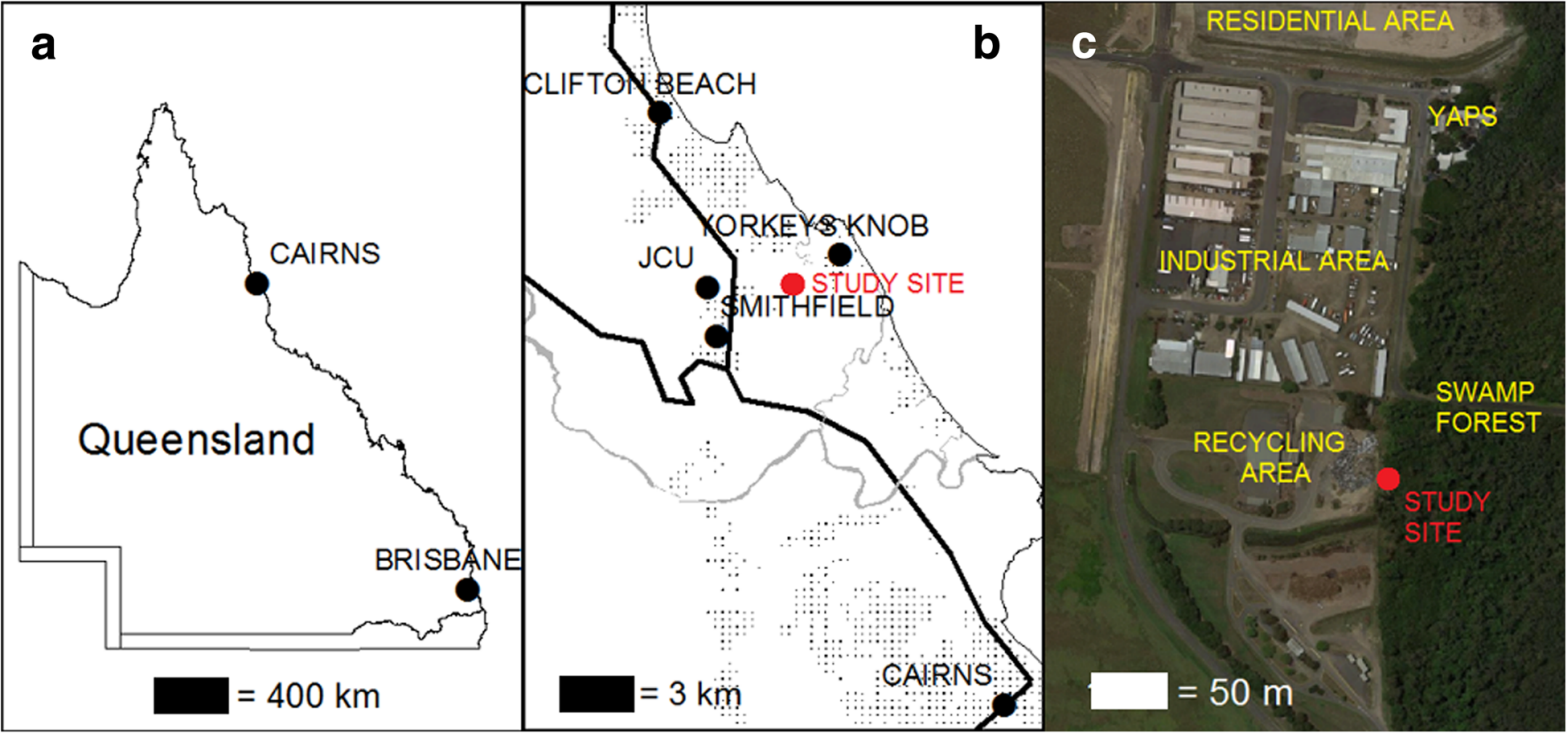
Map of Queensland (**a**) showing Cairns and Smithfield (**b**) as well as the layout of the Young Animal Protection Society (YAPS) study site (**c**)

The study was conducted in Smithfield, 15 km north of Cairns, Queensland, Australia (16.8221°S, 145.6972°E) (Fig. 1). The study site is situated proximal to an extensive swamp that provides larval habitats for a range of mosquito species near the Young Animal Protection Society (YAPS) Animal Refuge and the Smithfield Recycling Transfer Station. The specific location borders the recycling station and swamp forest with a tree canopy dominated by *Melaleuca*, *Archontophoenix* palms and *Ceriops* mangroves [9]. The closest house was ~500 m away. The site has a tropical climate with hot, humid summers and cooler, drier winters. Average annual rainfall is 1992 mm and temperatures range between 23–31 °C in the summer and 18–26 °C in the winter [10]. Although malaria was eliminated from Australia in 1981 [11], the former dominant vector, *Anopheles farauti*, is a common mosquito in northern Australia.

### Barrier screen design and mosquito sampling

Barrier screens were constructed of 6 m straight lengths of 1.8 m high, high-density polyethylene (HDPE) UV stabilised shade cloth (Coolaroo® Gale Pacific Ltd, Melbourne, Australia) attached to poles and erected parallel to the swamp forest. On the side of the barrier screen opposite to the swamp, 150 g of dry ice was placed within a 2 litre cooler jug with four small holes to release CO_2_. This was then placed 1 m behind each barrier screen to simulate a blood meal source. Although not usual, the dry ice was used here to maximise collections on the barrier screens and to facilitate a more powerful direct comparison of the different construction attributes.

The impact of four basic barrier screen parameters on numbers of mosquitoes collected were evaluated individually as follows: shade cloth weight; shade cloth colour; construction design; and frequency of inspection. After the optimal shade cloth weight was determined by comparing different shade cloth weights of identical colour, that weight was then used to determine optimal colour. The optimal weight and colour was then used to evaluate optimal designs and inspection frequencies.

**Fig. 2:**
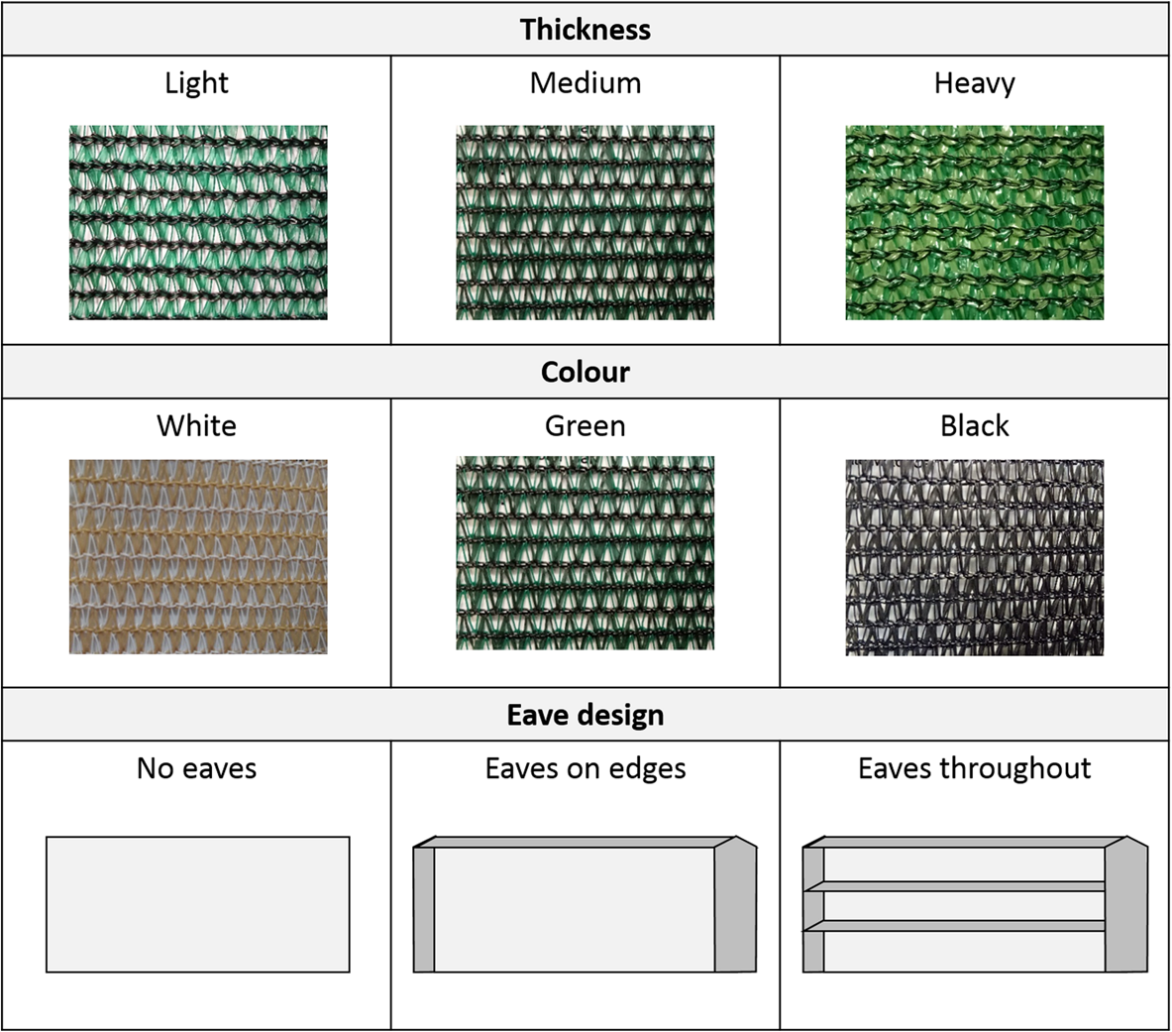
The factors that were evaluated to optimise the barrier screens included weight, colour and eave design

Cloth weight is defined by grams per square meter (g/m^2^) with green cloth of 135 g/m^2^, 160 g/m^2^ and 214 g/m^2^ tested, corresponding to 50%, 70% and 90% shading, respectively. The impact of colour was then evaluated using white, green and black cloths of optimal weight (determined as described previously). Barrier screen construction design was varied to determine if eaves of 25 cm depth could improve collection efficacy. It was hypothesized that mosquitoes would remain on the screens for longer periods by baffles or eaves; therefore, three barrier screen designs were evaluated using the optimal weight and colour cloth as previously determined. Screens without eaves (a straight 1.8 × 6 m flat shade cloth), screens with perimeter eaves (e.g. vertical eaves along both sides and a horizontal eave along the top) and a screen with complete eaves (e.g. vertical screens on both sides and three horizontal eaves at heights of 60 cm, 120 cm and 180 cm from the ground; Fig. 2). The frequency of inspections on mosquito numbers collected was evaluated by inspecting sets of identical screens (optimised for weight and colour as described above) at intervals of 30 min, 60 min and 90 min during 3-hour sampling periods.

A balanced Latin square design (3 × 3) was used to compare each of the experimental parameters. Triplicate barrier screens were erected in a row (separated by 2 m gaps). Each variable tested was rotated through all three potential spatial positions over three consecutive nights to eliminate any location associated bias (a full rotation of positions). For each variable three full rotations were completed unless specified otherwise.

Replicate barrier screens were examined for mosquitoes by a single collector from 18:00 h until 21:00 h using a mouth aspirator to remove resting mosquitoes from the screens. The collector applied mosquito repellent (active constituent 92.8 g/L Picaridin Aerogard®, Sydney, Australia) and unless stated otherwise, mosquitoes were collected hourly with each searching event lasting approximately 10 min per screen with the swamp side searched first. Mosquitoes from the same screen and hour were stored in separate labelled polyethylene terephthalate (PET) holding cups. The resting height on the screen, low (0–60 cm from the ground), middle (60–120 cm above the ground) or high (120–180 cm above the ground) was also recorded for 3 nights. All mosquitoes were morphologically identified to genera and sex [12]. The study was conducted between March 2016 and February 2018.

Cloth weight was tested over three rotations where light, medium and heavy cloth was compared, an additional 2 rotations with only light and medium weighted cloth (to ascertain if there was a significant difference in numbers of mosquitoes collected between light and medium cloth) was carried out. The influence of cloth colour on mosquito numbers was tested during four rotations over 12 nights: with white, green and black barrier screens.

Screens without eaves were initially compared to screens with perimeter eaves during 2 nights (1 rotation). During the initial testing period, differences between the two designs were not found so screens with complete eaves were added for an additional 2 rotations (6 more nights of testing). The impact of the frequency of inspection on mosquito numbers collected was tested over 9 nights (3 rotations with inspections at 30 min, 60 min and 90 min).

### Statistical analysis

The effect of barrier screen variables on resting female mosquito densities was analysed with a Generalized Linear Mixed Model (GLMM) with a negative binomial distribution and a random factor for the rotation of the Latin square (glmer.nb; package *lme4*) with a sequential *post-hoc* analysis to clarify any statistical differences between the experimental factors (glht; package *multcomp*). By incorporating the random factor for rotation into the GLMM, the model accounts for natural fluctuations in mosquito densities observed during sampling periods while increasing the power of the model. Separate analyses were conducted for each experimental parameter and for the *An. farauti* group (although *An. farauti* is the dominant species, *An. hinesorum* is found in the study area) as well as for mosquitoes in the genera *Aedes* and *Culex*. This analysis was conducted using R statistical software version 3.1.2.

## Results

A total of 6395 female unfed mosquitoes were captured over 24 nights. Of these 2668 were *An. farauti* (*s.l.*), 2807 were *Culex* mosquitoes (predominantly *Culex gelidus*, *Culex annulostris* and *Culex pullus*) and 920 were *Aedes* mosquitoes (made up of *Aedes vigilax* and *Aedes kochi*). Only 46 males were collected and blood-fed and gravid mosquitoes were not captured.

### Influence of cloth weight

**Fig. 3:**
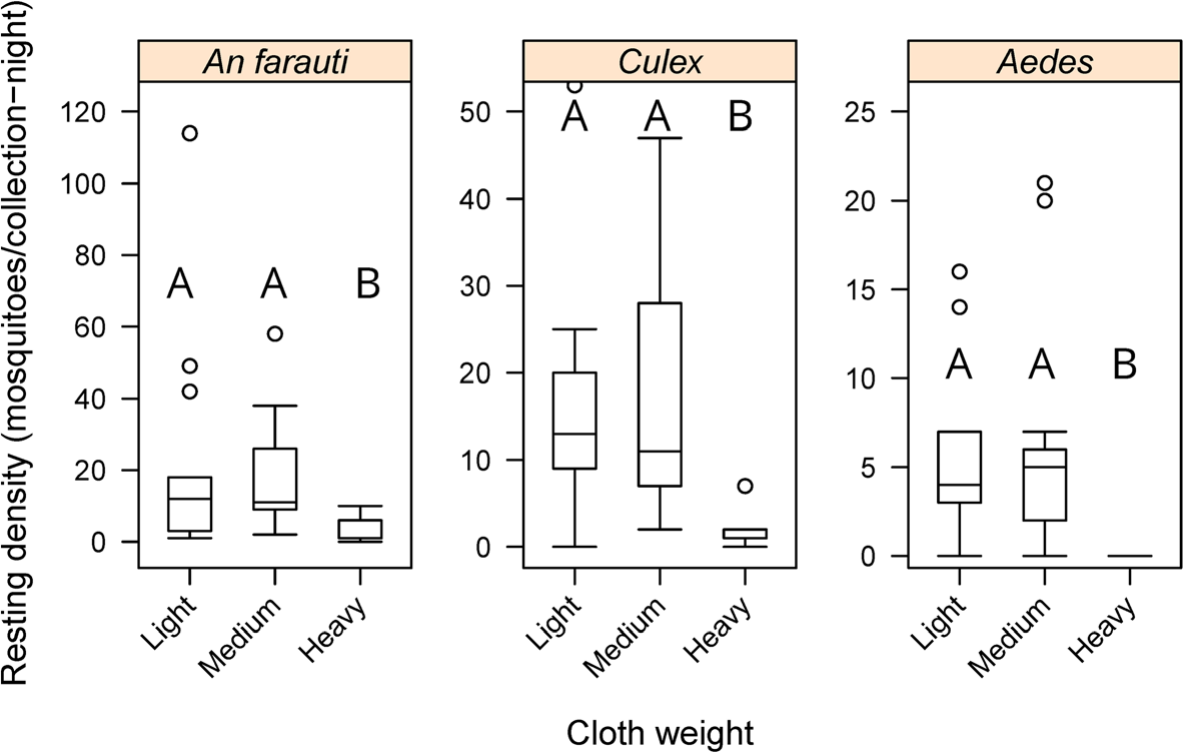
Comparison of the densities of female *Anopheles farauti*, *Culex* and *Aedes* mosquitoes caught resting on barrier screens with different weights. Different letters indicate significant difference (*P* < 0.05)

Cloth weight had an impact on the average number of female mosquitoes collected with fewer females captured on heavy netting (5 mosquitoes/collection-night (m/c-n)) compared to medium (43 m/c-n) or light cloth (43 m/c-n). Cloth weight significantly influenced the number of female *An. farauti* resting on the barrier screens (*β* = -0.7249, *SE* = 0.2591, *P* = 0.005) with the heavy shade cloth having fewer resting mosquitoes than barrier screens made with light and medium weighted shade cloth (Fig. 3 and Additional file 1: Table S1). The same effect of cloth weight was also found for *Culex* females (*β* = -0.7139, *SE* = 0.2253, *P* = 0.001) and *Aedes* females (*β* = -0.9555, *SE* = 0.2813, *P* = 0.0007).

### Influence of cloth colour

**Fig. 4:**
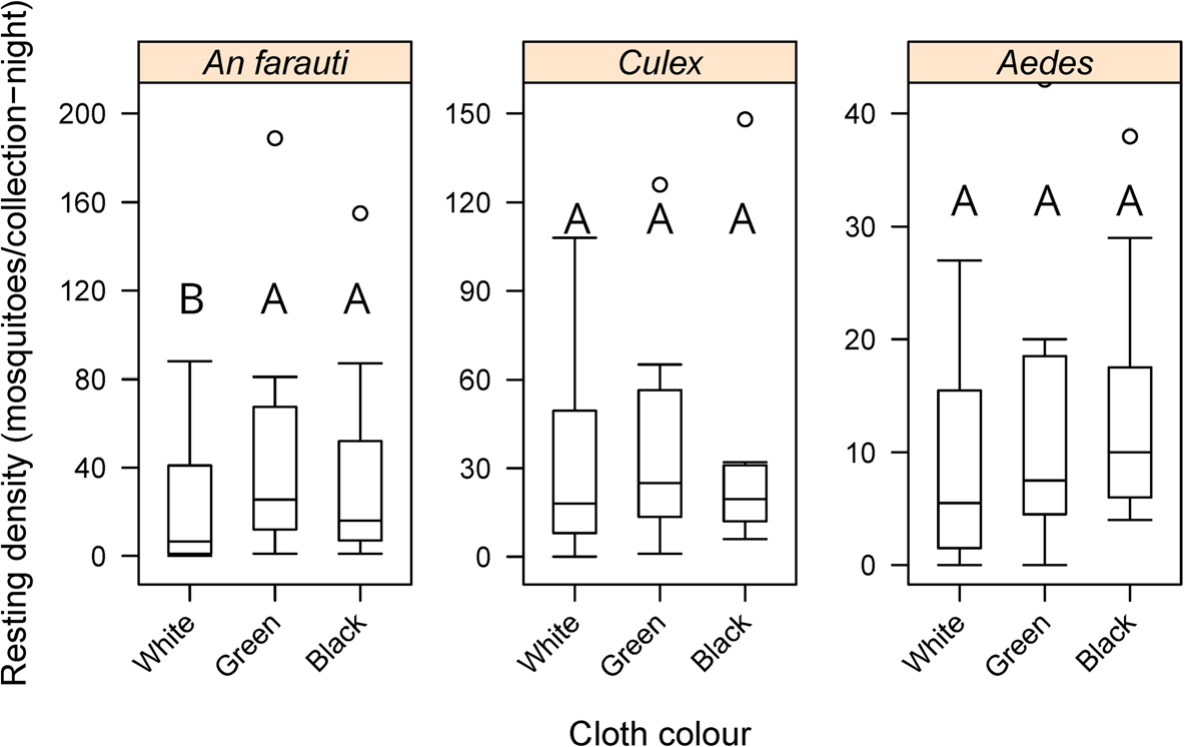
Comparison of the densities of female *Anopheles farauti, Culex* and *Aedes* mosquitoes caught resting on different colour barrier screens. Different letters indicate significant difference (*P* < 0.05)

The influence of cloth colour was compared using material of medium weight (160 g/m^2^). Cloth colour significantly impacted the average number of all female mosquitoes collected with fewer mosquitoes captured on white cloth (64 m/c-n) compared to green (118 m/c-n) or black cloth (103 m/c-n). Similarly, cloth colour significantly influenced the number of female *An. farauti* resting on the barrier screens (*β* = 0.3504, *SE* = 0.1757, *P* = 0.05) with the white colour having fewer *An. farauti* than green or black screens (Fig. 4 and Additional file 1: Table S1). Numbers of resting *Aedes* and *Culex* were not statistically significantly influenced by cloth colour (*β* = 2.1718, *SE* = 0.3879, *P* = 0.08; *β* = 0.2026, *SE* = 0.1723, *P* = 0.2, respectively).

### Influence of screen design (eaves)

**Fig. 5:**
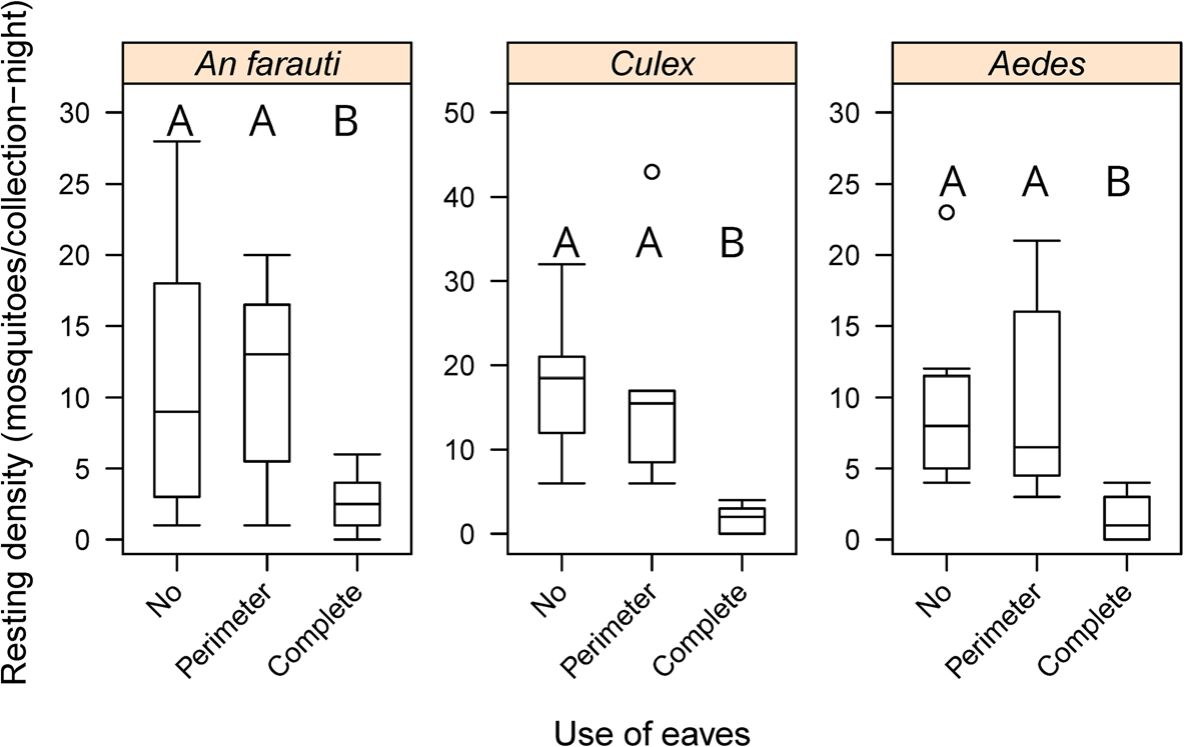
Comparison of the densities of female *Anopheles farauti*, *Culex* and *Aedes* mosquitoes caught resting on differing barrier screens designs: no, no eaves; perimeter, eaves on sides and top; and complete, 3 horizontal eaves and vertical eaves on the sides. Different letters indicate significant difference (*P* < 0.05)

The influence of screen design was compared using green material of medium weight. Eaves impacted the average number of all female mosquitoes collected with fewer mosquitoes captured on screens with complete eaves (6 m/c-n) compared to perimeter eaves (38 m/c-n) or no eaves (38 m/c-n). Similarly, the presence of eaves significantly influenced the average number of female *An. farauti* collected resting on the barrier screens (*β* = -0.4444, *SE* = 0.2021, *P* = 0.03), with the complete eave design having fewer mosquitoes captured on it than screens with perimeter eaves screens or screens without eaves (Fig. 5 and Additional file 1: Table S1). The same effect of eave design was also observed for *Culex* females (*β* = -0.9047, *SE* = 0.2419, *P* = 1.83e-04), and *Aedes* females (*β* = -0.5747, *SE* = 0.1891, *P* = 0.002).

### Influence of frequency of inspections

**Fig. 6:**
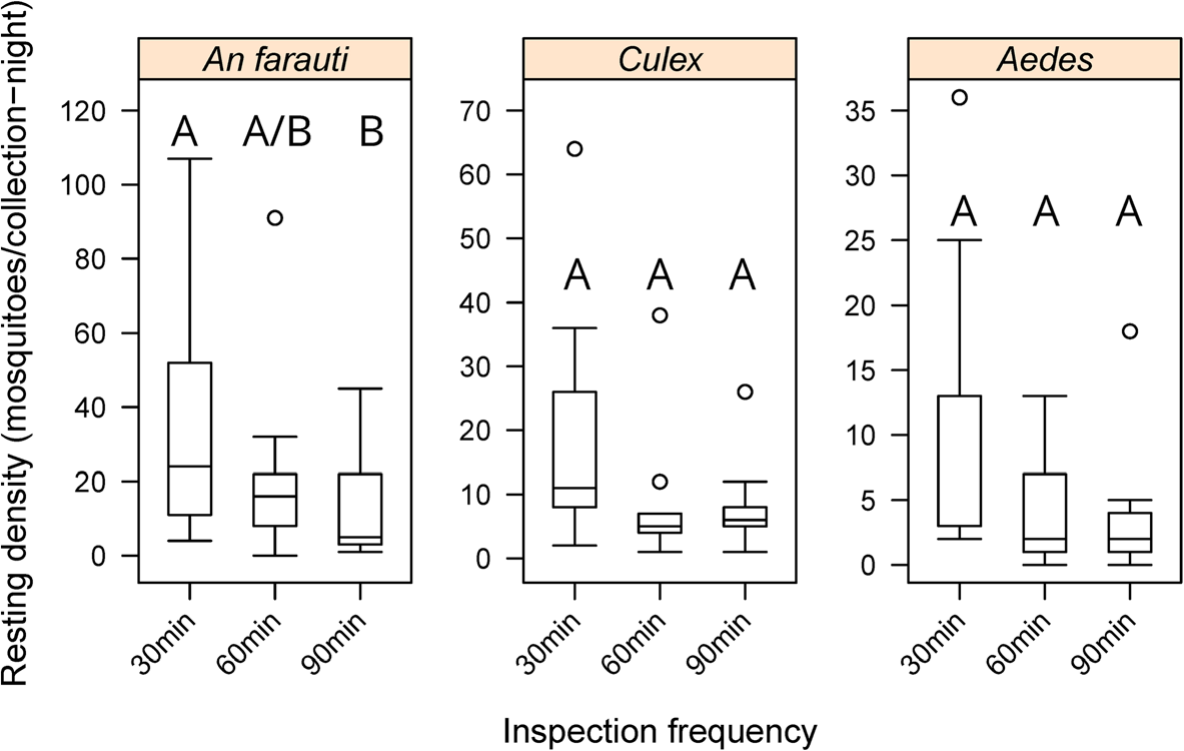
Comparison of the densities of female *Anopheles farauti*, *Culex* and *Aedes* mosquitoes caught resting on barrier screens with different intervals between inspection events. Different letters indicate significant difference (*P* < 0.05)

The influence of the frequency of inspections was compared using green material of medium weight and constructed without eaves. The average number of all female mosquitoes caught was inversely related to the length of time between inspections; inspections at 30 min intervals captured more mosquitoes (67 m/c-n) than inspections at 60 (36 m/c-n) and 90 min (26 m/c-n). Similarly, the length of the time period between inspections significantly influenced the average number of female *An. farauti* collected on the barrier screens, with inspections every 30 min collecting more resting *An farauti* than inspections every 60 and 90 min (*β* = -0.6268, *SE* = 0.1974, *P* = 0.001) (Fig. 6 and Additional file 1: Table S1). This pattern was also found for *Culex* resting females (*β* = -0.4500, *SE* = 0.1860, *P* = 0.01) and *Aedes* resting females (*β* = -0.5187, *SE* = 0.2183, *P* = 0.02).

### Comparison of resting heights

**Fig. 7:**
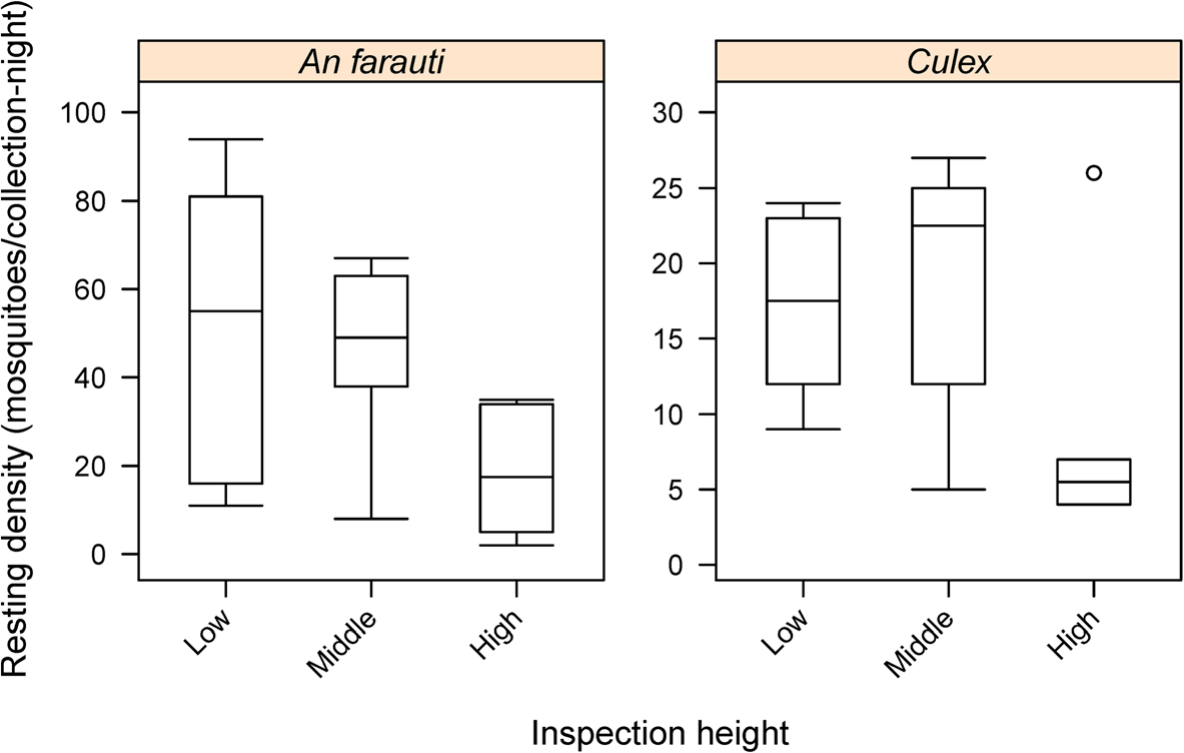
Comparison of the densities of female *Anopheles farauti*, *Culex* and *Aedes* mosquitoes caught resting on barrier screens on different resting heights: low, within 60 cm of the ground; middle, resting between 60 and 120 cm from the ground; and high, resting more than 120 cm above the ground

The average number of all female mosquitoes caught per night was fewer on the high (59 m/c-n) section than the middle (132 m/c-n) and low (142 m/c-n) sections. Similarly, the difference between heights of the barrier screen for resting *An. farauti* was significant with low and middle areas having more resting mosquitoes than the high areas (*β* = -0.5122, *SE* = 0.2237, *P* = 0.02) (Fig. 7). The same pattern of resting heights was also observed for *Culex* (*β* = -0.3301, *SE* = 0.1595, *P* = 0.04). Height of resting *Aedes* was not analysed statistically due to insufficient numbers.

## Discussion

The barrier screen is intended to be used in the field as a passive mosquito collecting method (without addition of a lure or attractant) to record natural mosquito densities and movements. As mosquito numbers in many locations are often low, a systematic assessment of some basic parameters of barrier screens were undertaken with a hypothesis that numbers of mosquitoes collected could be increased. While each experiment was designed to assess the impact of a single specific parameter on numbers of mosquitoes collected, it is important to note that all these factors interact and influence each other. The experimental design targeted host-seeking females by providing dry ice as a carbon dioxide source on one side of the screen to simulate the presence of a potential vertebrate host. The colour and density of screen material used as well as construction design and frequency of collection all significantly impacted the numbers of mosquitoes captured.

Fewer mosquitoes were collected resting on the heavy (90% - 214 g/m^2^) weight cloth; this may be due to the denser material limiting the amount of carbon dioxide passing through the heavy netting compared to the light and medium weight netting. Cloth material corresponding to 70% shading (160 g/m^2^) had comparable numbers of mosquitoes captured on it relative to the 50% (135 g/m^2^) shading. The 70% shade cloth would be advantageous for field use due to the increased strength and durability relative to 50% shade cloth.

Cloth colour was a significant factor influencing the effectiveness of barrier screens for collecting mosquitoes. Previously, darker barrier screens (black or green) were successfully used in the Solomon Islands and Papua New Guinea [8] whereas in Indonesia, black [8], white (N. Lobo, University of Notre Dame USA, 2018, unpublished data) and grey [13] barrier screens were used to collect mosquitoes. In general, mosquitoes prefer darker coloured substrates [14]. Anophelines are more frequently found in small resting boxes lined with darker fabrics than in boxes with lighter colours [15]. *Aedes* are also more attracted to darker coloured, lower reflective materials with black and red being more attractive than lighter yellows and whites [16–18]. Results from this study confirmed the observations of the original studies in Indonesia, Solomon Islands and Papua New Guinea, in that the higher colour contrast between the black screen and mosquitoes made it easier to see resting mosquitoes and thus capture them. Substrate reflectance may influence mosquito numbers collected and the physiological state of the mosquito may also affect preferences [14, 19].

It has been hypothesized that the frequency at which screens need to be inspected could be lessened if mosquitoes remained on the screens for longer periods by baffles or eaves; and preliminary data collected in Indonesia supports this hypothesis (N. Lobo, University of Notre Dame USA, 2018, personal communication). Hence, screens with eaves were created to test this hypothesis in Australia (e.g. the number of mosquitoes retained on barrier screens was hypothesized to be greater on barrier screens with eaves). However, the barrier screens with eaves in this experiment did not increase the number of resting mosquitoes collected. This may be due to the fact that resting mosquitoes could have been more difficult to see in edges or corners and/or the construction of the eaves described in this paper may function to make the screen thicker or denser (which was shown to be associated with fewer numbers of mosquitoes collected). The results from the experiments reported here suggest that simple linear barrier screens without eaves are recommended for, at least unfed, *An. farauti* collections, as eaves did not increase the numbers of mosquitoes collected despite the increased search time necessitated by the presence of eaves. Eaves also increased the likelihood of acquiring unwanted fauna such as spiders and associated webs. However, additional modifications to the design of barrier screens does deserve further attention, though the simplicity of the current design is one of its strengths.

All mosquitoes caught in these experiments were unfed and there was a significant increase in mosquitoes caught with more frequent inspections. Mosquitoes in different sex and physiological states might be expected to have different resting durations. While increasing the frequency of inspections to every 20 or even every 10 min might increase the numbers of mosquitoes collected, the increased presence of the collector inspecting the screen may serve as a lure to attract mosquitoes and thus could bias collection results. While topical repellent applied to the collector will prevent mosquito bites, the body heat and CO_2_ of the collector will act as attractants to mosquitoes and could lure mosquitoes to the vicinity of barrier screens. Based on our results, a collection interval of 30 min is recommended for collectors using a recommended topical repellent. However, this can be adapted based on the physiological status of mosquitoes being targeted for collection; therefore, if blood-fed mosquitoes are the focus of mosquito sampling, inspection events every 60 min might be sufficient, but this will require evaluation to confirm.

More mosquitoes were collected resting below 1.2 m than above it. Using suction traps in Africa, most mosquitoes (80%) were collected within a metre of the ground with catch number decreasing with increased height [20]. In Java, median nocturnal indoor resting heights of anophelines were measured: *An. aconitus*, *An. subpictus*, *An. indefinitus* rest within 38 cm of the floor while *An. kochi* was found within 68 cm of the floor [21] which agrees with the outdoor resting heights observed in this study for *An. farauti*. Using the barrier screens in Amazonian Peru most *An. darling* were collected less than 1m from the ground [22] which is similar to the results found in this study. There is limited data regarding resting and flying heights of mosquitoes and the barrier screen does provide a simple method for collecting such useful information. Barrier screens of increased height (up to 3 m) do deserve further attention to see if mosquitoes fly and rest at higher altitudes; however, this may also increase the difficulty in collecting mosquitoes from the barrier screens.

Since the barrier screen method was published in 2013, several studies have used this method to collect a combination of outdoor resting blood-fed, unfed, gravid and sugar-fed mosquitoes. The original barrier screen collections in Indonesia, the Solomon Islands and Papua New Guinea by Burkot et al. [8] used black or green screens without eaves of 70% shading weight, that were checked every 60 min. Russell et al. [23] also used green 70% shading weight screens in the Solomon Islands. Moreno et al. [22] in Amazonian Peru successfully captured *An. darlingi* using green, ‘lightweight’ barrier screens checked every 60 min. In Papua New Guinea, Keven et al. [24] used green, 70% shading without eaves and checked their screens every 20 min. The results of the experiments reported here suggest simple (without eaves) barrier screens at least 120 cm tall constructed with black 70% shading weight netting can effectively be used to collect mosquitoes with collections every 30 min. Further experiments are recommended that would record mosquito behaviour upon encountering the barrier screen and to visualize initial responses to answer questions such as how long mosquitoes of different species, sex and physiological states rest on the barrier screen.

## Conclusions

The barrier screen is a relatively new adaptable tool that can answer a number of behavioural questions relevant for the surveillance and basic understanding of vectors by sex or physiological status. Data collected by barrier screens can then be translated to inform control strategies. Although publications from four countries report data collected using the barrier screen, this is the first paper seeking to maximise the barrier screen method. Barrier screens were developed to collect anophelines outdoors, and this method has demonstrated diversity in its capability to collect a wide range of other mosquito species as well as flexibility and compatibility in the numbers and locations in which barrier screens can be deployed to explore mosquito movements within rural and domestic environments.

## Additional file

Additional file 1: **Table S1.** *Post-hoc* comparisons of the densities of females *An. farauti*, *Culex* and *Aedes* mosquitoes caught resting on the barrier screens between each experimental factors (asterisks indicate strength of significance above *P* > 0.05). (PDF 123 kb)

## Abbreviations

GLMM: Generalized linear mixed model
HBI: Human blood index
HDPE: High-density polyethylene
m/c-n: Mosquitoes per collection night
PET: Polyethylene terephthalate
SE: Standard error
YAPS: Young Animal Protection Society
