# Supplementary material for: Maximising mosquito collections from barrier screens: the impacts of physical design and operation parameters
Source: Parasit Vectors. 2019 Jan 14;12:31. doi: 10.1186/s13071-019-3291-4 (PMC6332603; doi:10.1186/s13071-019-3291-4)
Supplement: Supplementary file 1 — Table S1. Post-hoc comparisons of the densities of females An. farauti, Culex and Aedes mosquitoes caught resting on the barrier screens between each experimental factors (asterisks indicate strength of significance above P > 0.05). (PDF 123 kb) [file 13071_2019_3291_MOESM1_ESM.pdf]

Table 1. Additional file 1

Mosquito group	Experimental factors	Parameter Variable	Test values
An farauti	Weight	Medium – Light	(β = 0.1775, se = 0.3173, p = 0.84)
		Heavy – Light	(β = -1.8271, se = 0.4070, p = 1.9e-05)***
		Heavy – Medium	(β = -2.0046, se = 0.4125, p = < 1e-05)***
	Colour	Green – White	(β = 1.0615, se = 0.3022, p = 0.0013)**
		Black – White	(β = 0.7271, se = 0.2928, p = 0.035)*
		Black – Green	(β = -0.3344, se = 0.2881, p = 0.48)
	Design	Perimeter – None	(β = 0.0703, se = 0.2435, p = 0.95)
		Complete – None	(β = -1.2207, se = 0.3542, p = 0.0015)**
		Complete – Perimeter	(β = -1.2910, se = 0.3561, p = 0.00075)***
	Search frequency	60min – 30min	(β = -0.7285, se = 0.3871, p = 0.14)
		90min – 30min	(β = -1.2519, se = 0.3987, p = 0.0048)**
		90min – 60min	(β = -0.5235, se = 0.3917, p = 0.37)
Culex	Weight	Medium – Light	(β = 0.0765, se = 0.2643, p = 0.95)
		Heavy – Light	(β = -1.8854, se = 0.3857, p = 2.2e-06)***
		Heavy – Medium	(β = -1.9619, se = 0.3866, p = < 1e-06)***
	Colour	Green – White	(β = 0.5665, se = 0.3206, p = 0.18)
		Black – White	(β = 0.4353, se = 0.3196, p = 0.36)
		Black – Green	(β = -0.1312, se = 0.3056, p = 0.90)
	Design	Perimeter – None	(β = -0.0736, se = 0.2578, p = 0.95)
		Complete – None	(β = -2.2632, se = 0.3981, p = < 1e-06)***

		Complete – Perimeter	(β = -2.1896, se = 0.3987, p = < 1e-06)***
	Search frequency	60min – 30min	(β = -0.8294, se = 0.3777, p = 0.72)
		90min – 30min	(β = -0.8862, se = 0.3785, p = 0.050)
		90min – 60min	(β = -0.0568, se = 0.3888, p = 0.98)
Aedes	Weight	Medium – Light	(β = 0.0373, se = 0.1503, p = 0.96)
		Heavy – Light	(β = -1.5112, se = 0.3605, p = < 1e-04)***
		Heavy – Medium	(β = 1.5485, se = 0.3599, p = < 1e-04)***
	Colour	Green – White	(β = 0.4250, se = 0.2523, p = 0.21)
		Black – White	(β = 0.5529, se = 0.2539, p = 0.075)·
		Black – Green	(β = 0.1278, se = 0.2447, p = 0.86)
	Design	Perimeter – None	(β = -0.0159, se = 0.2225, p = 0.99)
		Complete – None	(β = -1.5593, se = 0.3965, p = <0.001)***
		Complete – Perimeter	(β = -1.5434, se = 0.3985, p = 0.001)***
	Search frequency	60min – 30min	(β = -0.7008, se = 0.4357, p = 0.24)
		90min – 30min	(β = -1.0232, se = 0.4407, p = 0.053)
		90min – 60min	(β = -0.3223, se = 0.4612, p = 0.76)
